# Why did children grow so well at hard times? The ultimate importance of pathogen control during puberty

**DOI:** 10.1093/emph/eov017

**Published:** 2015-07-20

**Authors:** Peeter Hõrak, Markus Valge

**Affiliations:** ^1^Department of Zoology, Institute of Ecology and Earth Sciences, Tartu University, Vanemuise 46, 51014, Tartu, Estonia;; ^2^Institute of Psychology, Tartu University, Näituse 2, 50409, Tartu, Estonia

**Keywords:** epidemiological transition, Flynn effect, height, infant mortality, pubertal growth spurt, secular trend

## Abstract

**Background and objectives**: Secular increase in human height and performance occurred in Europe throughout the 20th century despite the temporally worsening access to nutrients during and after World War II. This pattern is paradoxical under the assumption of the major impact of pre- and postnatal growth conditions for determination of adult size and human capital.

**Methodology**: We examined the anthropometric parameters of Estonian girls born between 1938 and 1953, and measured around the age of 17 (*n* = 1475). This period involved two opposite trends in the economic and epidemiological situation: increasing birth-time economic hardships during the war and particularly in the post-war period, and decreasing infant mortality (a proxy of disease burden) after 1947.

**Results**: Height of girls was negatively affected by the number of siblings and positively by parental socioeconomic position, but these effects were weaker than the secular trend. Leg length (an indicator of pre-pubertal growth conditions) was independent of age and birth date while all other traits, including measures of performance (cranial volume, lung capacity and handgrip strength) showed acceleration. The best predictor of size at age 17 was, in most cases, infant mortality in the year when the girls were aged 11.

**Conclusions and implications**: Reduction of disease burden during pubertal growth can override effects of resource shortage at birth. Our results also support the idea that increasing efficiency of pathogen control can contribute to the secular increase in cognitive abilities, i.e. the Flynn effect, and that epidemiological transition is the main driver of secular increase in human capital.

## INTRODUCTION

Resource limitation and pathogens are arguably the most important selective forces that affect fitness via survival and fecundity selection [1]. These effects are often mediated via ontogenetic modifications of adult phenotype [2, 3] and their relative importance varies both spatially and temporally. For instance, nutritional/socioeconomic factors versus disease burden seem to contribute differentially to secular trends in human height on different continents [4] and even within different parts of Europe and cohorts within countries [5, 6]. Such studies on human ontogeny have had an impact on the advancement of life-history theory in general (e.g. Refs. [3, 7, 8]) and also carry applied value in fields as diverse as epidemiology [8, 9], paediatrics [10, 11], evolutionary medicine [7, 12, 13], cognitive science [14, 15] and economic [6, 16] and cultural [17] history.

European history during the 20th century offers a unique setting for studying the simultaneous effects of resource limitation and pathogen control on human growth. Inhabitants of countries involved in World War II (WWII) experienced a rapid decline in living standards during the war. Deterioration of living conditions was particularly strong during the post-war period in areas under the rule of the communist regime [18, 19]. At the same time, improvements in medical care, based much on the introduction of sulphonamide drugs, antibiotics and vaccination programmes, contributed to the decline in mortality due to infectious diseases after the 1940s [5]. These improvements in the disease environment are often considered to be the most important drivers of the secular increase in human height [4, 5] because fighting infections reallocates resources that could be otherwise invested into somatic growth (e.g. Refs. [13, 20]) and because intestinal infections inhibit the absorption of nutrients [9].

**Figure 1.**
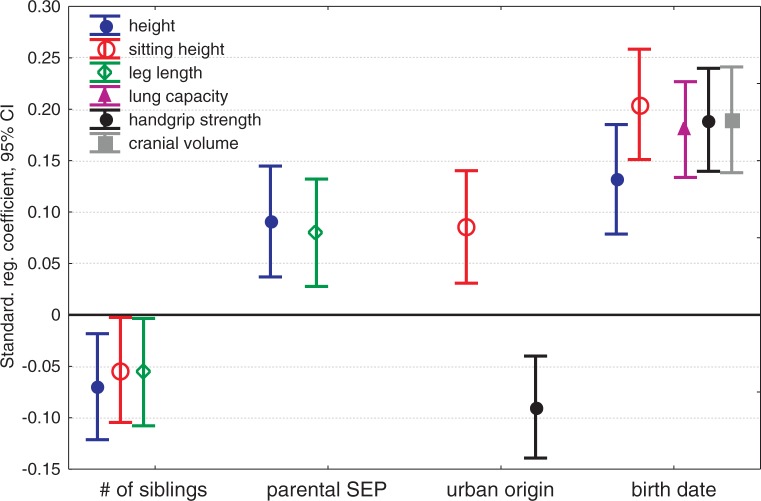
Standardized regression coefficients (β ± 95% CI) for significant predictors of measured parameters of size and performance. β-s are from models presented in Table 1

Here, we use data from 1475 Estonian adolescent girls to assess the effects of resource limitation and the epidemiological situation on various anthropometric traits. The dataset, collected by Prof. Juhan Aul (1897–1994), includes girls born between 1938 and 1953, i.e. during the period involving a highly variable epidemiological situation at the time of birth. Infant mortality increased rapidly from 1944 and started to decline from 1948, dropping below the pre-war level by 1952 (Fig. 1E). Infant mortality is considered a relevant proxy for the prevalence of disease [4] and particularly infection and inflammation [20]. The girls born between 1943 and 1947 can thus be considered to have experienced harsher pre- and postnatal disease conditions than those born before and after that period. On the other hand, their period of pubertal growth coincided with a more relaxed pathogen stress than compared with the girls born before the war (Fig. 1E). More interestingly, the post-war decline in infant mortality coincided with the rapid deterioration of living standards after 1947, which was partly caused by the destruction of the rural economy by mass-collectivization [18]. Culmination of the health crisis in post-war Estonia is indicated by the absolute number of recorded deaths that peaked in 1946–47, a pattern uncommon in the rest of Europe [21]. The study period thus involved two opposite trends in the economic and epidemiological situations: increasing birth-time economic hardships during the war and particularly in the post-war period, and decreasing infant mortality after 1948.

Such a setting offered us an opportunity to compare the effects of resource availability and pathogen control on human growth. Furthermore, these data enabled us to distinguish between the external effects on early postnatal and pubertal growth. Because legs grow faster than the trunk before puberty, leg length may capture exposures earlier in life than trunk length [22–24] (but see Ref. [25]). In addition to measures of stature we tested for the presence of a secular trend and the effect of resource limitation on cranial volume, lung capacity and handgrip strength. These variables can be considered as proxies for current and future performance and hence, fitness. Cranial volume correlates with cognitive ability [26, 27] and it has been hypothesized that secular trends in head size may be responsible for the long-sustained increase in IQ scores during the 20th century [14]. The causes of this phenomenon, known as the Flynn effect are still poorly understood [15, 28]. Lung capacity is positively associated with birth weight [10], is negatively affected by exposures throughout life and is indicative of pulmonary function that offers protection from respiratory [29] and cardiovascular [30] disease. Handgrip strength is a measure of physical health that is negatively correlated with disability, morbidity and mortality rates in adults. It is also indicative of blood testosterone levels and levels of fat-free body mass [31]. We asked how these measures of performance, that are potentially related to fitness, are affected by growth conditions.

Under the hypothesis that all measured traits are sensitive to pre- and postnatal growth conditions [4, 5, 22], we predicted that the girls born in the middle of our study period were smaller and weaker than those born before the war and after 1948. Under this scenario, anthropometric traits would correlate most strongly with neonatal mortality in the birth year. Alternatively, if pubertal growth appears more sensitive to external conditions than pre- and postnatal growth, we should detect an increasing trend in all studied variables. Under this scenario, anthropometric traits would correlate most strongly with neonatal mortality in the year that girls enter the most rapid phase of pubertal growth, i.e. at age 11 [19, 32]. We also asked which of the measured traits are most sensitive to external effects. In particular, we expected to see the strongest effects of postnatal growth conditions on leg length [22] and lung capacity [10].

## METHODOLOGY

### Historical and socioeconomic background of the study

The settlement of Estonia by Finno-Ugric people started around 8500 BC. Having been under Danish, German and Swedish rule from the 13th century, Estonia became part of the Russian Empire in 1710. In 1819, serfdom was abolished, allowing the peasants to own their own land or move to the cities. These moves created the economic foundation for the Estonian national cultural awakening in the second half of 19th century. With the collapse of the Russian empire in World War I, Estonia became independent after winning a war against Soviet Russia in 1920. During the following post-war period the country prospered; for instance the GDP per capita in 1938 was comparable to that of Czechoslovakia and Hungary [33]. The Republic of Estonia was occupied by the Soviet Union in June 1940. In June 1941, the Soviet authorities organized the first mass deportations, targeting the elite; ∼10 000 people were sent to prison or labour camps in Russia. In July 1941 Germany invaded quickly. German occupation on the mainland lasted until September 1944 with the Soviets taking over again. Total WWII population losses in Estonia were estimated among the highest proportion in Europe [18]. Altogether, out of a population of 1.1 million, ∼47 000 were arrested for political reasons and 35 000 were deported during the period of Stalinist repression [34]. The terror of the first Soviet year motivated ∼72 000 Estonians to flee to the west when the return of the Soviet regime became obvious. The second mass deportation took place in March 1949. Shortly afterwards, the establishment of collective farms (kolkhozes) was announced and the majority of peasants joined, fearing that they would be deported if they did not sign up [34]. With collectivization, output, productivity and the level of mechanization declined in Estonian agriculture, especially after 1951. During the 1950s, masses of farm animals would starve to death in late winter or early spring because of a lack of fodder. Overall agricultural production declined to less than half of the pre-war level and, for many peasant families, the years after collectivization were the poorest of their lives. Private production on garden plots owned by the entire rural population and a part of the urban population remained very important [35]. The situation in the countryside improved with increasing state subsidies for agriculture beginning in the 1960s (http://www.estonica.org/en/Collectivization_of_Estonian_Agriculture/, 29 July 2015, date last accessed).

### Data

Anthropometric data from 1752 schoolgirls, born between March 1938 and November 1953 were collected between January 1956 and September 1969 by a single person, Juhan Aul. Along with anthropometric measures, number of siblings, place of residence and parental occupations were recorded. Because the later dataset was incomplete, we were able to use the records from 1475 girls in this study. Their average age at measurement was 16.85 ± 0.88 (SD) years, ranging from 14.6 to 20.0 years. Anthropometric variables were recorded according to Martin and Saller [36]. Leg length was calculated by subtracting sitting height (trunk length, see Fig. 4 in Ref. [23]) from the total height. Vital capacity of lungs was measured using a bellows type spirometer. Maximum handgrip strength was registered with a handheld dynamometer. If data for handgrip strength were available for both hands, the highest measure was used. Cranial volume was calculated according to the formula: (7.884*(head length −11)+10.842)*((head width −11) −1593.96))); units in mm [37]. Data for infant mortality (i.e. mortality between birth and 1 year of age) were obtained from Estonian Population Register (http://pub.stat.ee/px-web.2001/I_Databas/Population/databasetree.asp, 29 July 2015, date last accessed). This database lacked figures for 1941, 1943 and 1944). We obtained archive data for the first half of 1943 and derived the yearly infant mortality for 1943 from these. For calculations involving infant mortality at birth year we obtained figures for 1941 and 1944 by averaging the corresponding figures of two adjacent years. We had complete original data for calculations involving infant mortalities in those periods where the girls were 11 years old (variable MORT11). Parental socioeconomic position (SEP) was divided to four classes: (i) unskilled manual workers, (ii) skilled manual workers, (iii) professions requiring secondary education and (iv) professions requiring tertiary education. If mother and father differed in their SEP rank, the highest was used.

### Statistics

**Figure 2.**
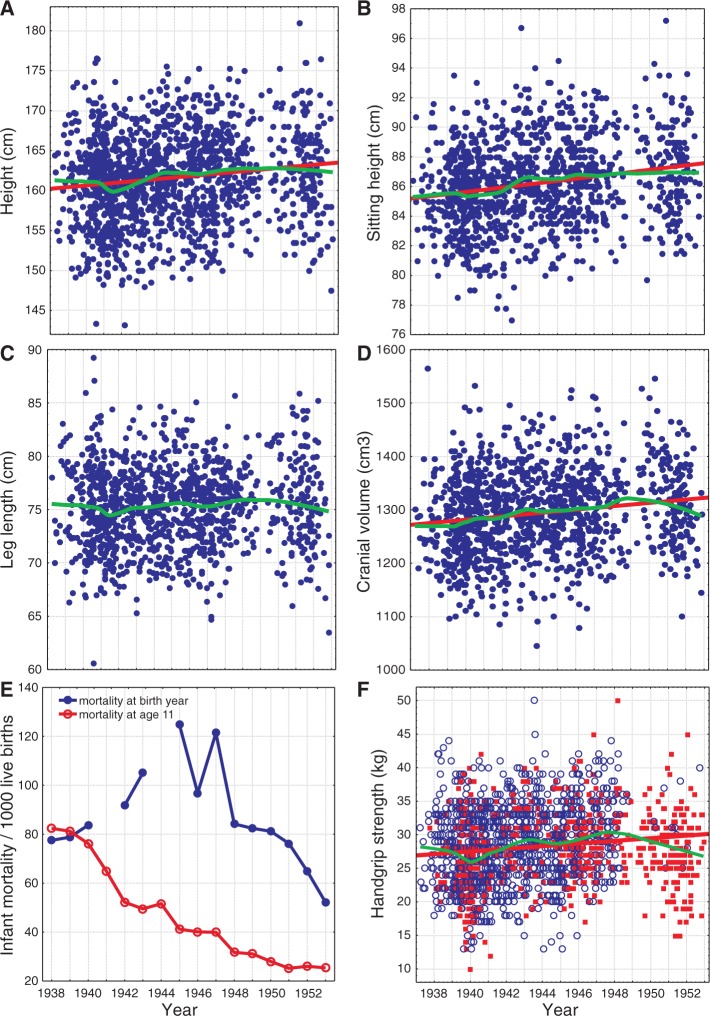
Secular trends in height, cranial volume and handgrip strength (**A–D**, **F**) and trends in infant mortality (**E**). Straight Lines are for linear regressions, unadjusted for covariates in Table 1. Splines are generated by LOWESS smoothing. In (F), open symbols denote rural and closed symbols urban origin

**Figure 3.**
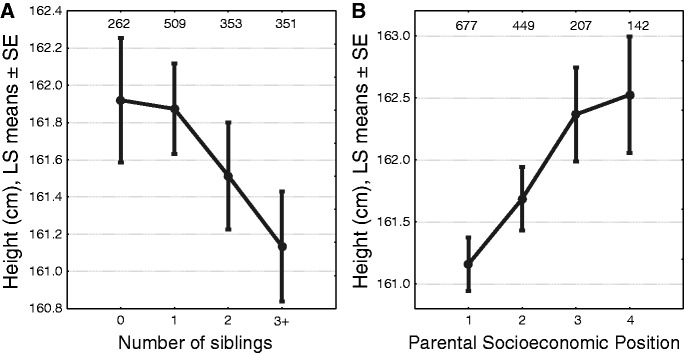
Effects of the number of siblings (A) and parental SEP (B) on height. LS means adjusted for significant covariates, indicated in Table 1

All the anthropometric variables were normally distributed. We used multiple regressions for testing the simultaneous effects of birth date, number of siblings, parental SEP, age and urban versus rural residence (as a dummy variable). In Fig. 3, number of siblings and parental SEP are entered as factors into ANCOVA models in order to calculate least square means accounting for other significant predictors. Non-significant predictor variables were dropped from the final models one at time. To enable comparison of the effects of different predictors on studied variables, we present standardized regression coefficients for significant predictors (β-s) along with 95% CI in Table 1 and Fig. 1. These refer to how many standard deviations a dependent variable will change, per standard deviation change in the predictor variable. In models involving lung capacity and handgrip strength, body mass was used as a covariate. In order to detect possible non-linear time trends in anthropometric variables, we applied locally weighted (LOWESS) smoothing on scatterplots presented in Fig. 2. All tests are two-tailed with a *P*-level below 0.05 as a criterion for significance.

**Table 1.** Predictors of anthropometric parameters of adolescent girls in multiple regressions

Trait	**Height**			**Sitting height**			**Leg length**		
Effect	*F*	*P*	β (SE)	*F*	*P*	β (SE)	*F*	*P*	β (SE)
Siblings	5.4	0.020	−0.07 (0.03)	3.4	0.063*	−0.05 (0.03)	3.7	0.054**	−0.06 (0.03)
SEP	6.8	0.009	0.09 (0.03)	2.3	0.134		6.7	0.010	0.08 (0.03)
Urban	2.5	0.111		5.8	0.016	0.09 (0.03)	0.2	0.645	
Birth date	18.3	0.00002	0.13 (0.03)	52.2	<0.00001	0.20 (0.03)	0.4	0.505	
Age	21.0	<0.00001	0.12 (0.03)	47.9	<0.00001	0.18 (0.03)	1.8	0.182	

Trait	**Cranial volume**			**Lung capacity**			**Handgrip strength**		
Effect	*F*	*P*	β (SE)	F	p	β (SE)	F	P	β (SE)
Siblings	1.1	0.288		0.4	0.527		0.2	0.638	
SEP	3.4	0.068		3.7	0.053		2.6	0.108	
Urban	0.7	0.390		0.1	0.799		6.7	0.009	0.09 (0.03)
Birth date	41.3	<0.00001	0.19 (0.03)	42.6	<0.00001	0.18 (0.02)	57.8	<0.00001	0.19 (0.03)
Age	14.6	0.0001	0.09 (0.03)	0.1	0.811		28.0	<0.00001	0.14 (0.02)
Mass		not tested		222.9	<0.00001	0.37 (0.02)	225.6	<0.00001	0.36 (0.02)

*n* = 1475 for all variables. *F*- and *P*-values are given for full models, standardized regression coefficients (β) are from final models containing only significant predictors. SEP: parental SEP, urban denotes urban versus rural origin. *The effect of siblings becomes significant (*F* = 4.2, *P* = 0.040) when parental SEP is removed from the model. **The effect of siblings becomes significant (*F* = 4.4, *P* = 0.037) when all other predictors except SEP are removed from the model.

## RESULTS

All studied anthropometric measures except leg length increased with birth date between 1938 and 1953 (Table 1 and Fig. 1). Leg length was also not affected by age at the time of measurement. Among most other traits (except body height), the magnitude of increase was similar: ∼0.2 SD units per SD unit of the 16-year study period. That is, most of the measured traits increased ∼0.12 SD units per decade. Unadjusted values for height increased by 4 cm during the study period (2.5 cm per decade; Fig. 2A) while sitting height increased by ∼2.5 cm (1.25 cm per decade; Fig. 2B). Cranial capacity increased ∼40 cm^3^ during the study period (25 cm^3^ per decade; Fig. 2D). The traits studied had different sensitivities to parental and family influences. The number of siblings negatively affected all three measures of height but none of the other variables (Table 1 and Fig. 1). Girls from families with one or two children were on average 0.8 cm taller than girls with three or more siblings (Fig. 3A). Parental SEP had a positive effect on two height measures—total and leg length. Girls from families with the lowest SEP (parents were unskilled manual workers) were on average 1.3 cm shorter than those girls whose parents worked in professions requiring tertiary or secondary education (Fig. 3B). Urban girls had on average 0.5 cm longer trunks than rural girls (LS means adjusted for the covariates in Table 1). Urban girls were weaker than those of rural origin. Cranial and lung volumes were not affected by family size, parental SEP or urban versus rural origin. Lung capacity was also independent of age at the time of measurement.

In order to test whether the secular trend in cranial volume was independent of an allometric increase in body dimensions, we examined whether the effect of birth date on head size remains significant in models containing either height, body mass or both as covariates. In all cases the effect of birth date on cranial volume remained significant (*P* < 0.0000001; β = 0.13 … 0.16).

Infant mortality in birth year did not correlate with year of measurement (*r* = −0.31, *P* = 0.277, *n* = 14; Fig. 2E) or with infant mortality in the year when the girls were 11 years old (MORT11) (*r* = −0.10, *P* = 0.746, *n* = 14; Fig. 2E). MORT11 decreased throughout the study period from 1949 to 1964 (*r* = −0.96, *P* < 0.0001, *n* = 16). Because of this multicollinearity, birth date and MORT11 were never simultaneously significant when entered in the models in Table 1. In most cases entering the MORT11 into models in Table 1 cancelled the effect of birth date on dependent variables (see Supplementary Information). In such cases standardized regression coefficients were of similar magnitude as in the case of birth date (height: β = −0.14 ± 0.03 (SE); sitting height: −0.21 ± 0.03; cranial capacity: −0.19 ± 0.03; handgrip strength −0.19 ± 0.02). Leg length was insensitive to MORT11 (F_1,1471_ = 1.2, *P* = 0.278) and infant mortality in birth year (F_1,1471_ = 0.4, *P* = 0.542). Lung capacity was a single variable with a dissimilar pattern. When the model included birth date, neither MORT11 nor infant mortality significantly predicted lung capacity. When birth date was removed, both indices of mortality became significant. Furthermore, the effect of MORT11 was stronger (β = −0.17 ± 0.02 (SE)) than that of infant mortality at birth year (−0.06 ± 0.02). In none of the other models did infant mortality in birth year affect any of the measured variables (*P* = 0.1 … 0.8). Absence of covariation between infant mortality in birth year and anthropometric traits can be also detected by observation of LOWESS splines in Fig. 2.

## DISCUSSION

### Pubertal versus early growth

Humans grow most rapidly during the first 2 years and during puberty. These growth periods are under different hormonal controls, which underlie differential trade-offs with immune function and nutritional sensitivities of early postnatal versus later growth [38]. In the current dataset, the pubertal growth spurt occurs between the ages of 10 and 12 [32]. Previous extensive studies worldwide (e.g. Refs. [10, 39, 40]) have predominantly emphasized the major impact of foetal and post-neonatal growth condition upon adult size, health and human capital. The importance of adolescent period has been recognized just recently [41]. Our data clearly show that all studied anthropometric traits (with the exception of leg length) were more sensitive to growth conditions in the 11th year of age than to conditions in the year of birth. This finding compares favourably with patterns of cohort specific-mortality In Sweden, France, Switzerland and England during the 18th and 19th centuries [20], where most temporal variance in old-age mortality for any cohort was related to variance in mortality during adolescence, i.e. between ages 10 and 14. Notably, infant mortality of a cohort was a much weaker predictor of old-age mortality than adolescent mortality of the same cohort (Table 1 in Ref. [20]). Such a pattern can be interpreted as evidence hinting that infections encountered during adolescence can have a stronger health impact than infections encountered in infancy.

Our measure of growth conditions—infant mortality rate for a given year—is considered as a highly relevant proxy for pathogen load [4, 16, 20]. Epidemiological studies of the links between height and diseases point to the importance of (repeated) respiratory and gastrointestinal microbial infections (reviewed by Refs. [4, 5, 42, 43]). These infections, which are common to infants and young children, restrict the body’s ability to absorb nutrients and also trigger metabolically demanding immune responses. Studies of UK birth cohorts have found that serious illnesses during childhood can reduce adult height by a 1–2 cm (reviewed by Ref. [5]). In the 1990s, 6-year old children in Russia were on average 2 cm taller in communities with a hospital on site [44]. As reviewed by Hatton [5], many of the landmark medical discoveries of the 20th century become available around WWII. Streptomycin (effective against tuberculosis) was introduced in 1947, sulphonamides and sulphapyridine (effective against bronchitis, pneumonia and influenza and whooping cough) after 1938 and antibiotics still later. Indeed, as we are reminded by Beard and Blaser [42], antibiotics are used as growth promoters in animal husbandry and therefore their effect on human growth should not be surprising.

We believe that these medical interventions, along with compulsory vaccination practices were primary drivers of the secular trend in measures of size and performance among the adolescent girls sampled in this study. For instance, the incidence of typhus dropped from 1 953 registered cases between 1946 and 1948 to 67 registered cases between 1947 and 1969 subsequent to the vaccination programme implemented between 1946 and 1950. The number of primary diagnosis cases of tuberculosis per year during the period 1946–47 was ∼4000; this number increased to 6000 during the period 1949–50 and started to decline subsequent to 1953 in parallel with the application of combined treatment with streptomycin and para-aminosalicylic acid. In 1960, 2670 primary cases of tuberculosis were registered while in 1970, this number had dropped to 879 (72 cases per 100 000) with further decline following [45]. Typhoid fever and Diphtheria peaked in 1945 and started to decline thereafter [46]. On the other hand, the prevalence of *Helicobacter pylori* infection, another suspected source of growth stunting [42], remained as high as 90% among the cohorts born before 1960 [47].

Our data suggest that the effects of improving medical conditions during the period of the pubertal growth spurt can be strong enough to override the preceding trend of worsening economic conditions during postnatal growth. In line with our findings, child height increased during WWII in most regions in England and Wales [48] and even in Moscow [16] despite severe rationing of consumables.

The pattern of secular increase of adolescent girls in this study is remarkably similar to that of adolescent boys in Poland, measured during the same period [49]. Poland experienced roughly similar political and socioeconomic shocks following the end of WWII, yet no reversals or stagnation of secular trend in height occurred. Bielecki *et al.* [49] suggest that the secular trends in height in post-war Poland could be at least partly subscribed to intensive rural to urban migration after 1955, leading to overall improvement in health and well-being and parental SEP. In Estonia, urban girls were not taller than rural ones (although they had taller trunks); yet the secular trend in all variables (except leg length) persisted after controlling for the urban versus rural origin and parental SEP of subjects (Table 1).

Similarly to Poland and Estonia, scarcity of food during and immediately after WWII did not stop the secular trend in height in urban centres of the Czech Republic and Novosibirsk in Russia [50]. Czechs introduced obligatory immunization schemes against communicable diseases, and regular obligatory preventive medical checks from birth to 18 years of age were implemented during the post-war period [19]. The Soviet health care system was claimed to be particularly effective at controlling infectious diseases [16]. Indeed, life expectancy at birth in Russia increased between 1946 and 1956 from 41.5 to 61.0 years in men and from 51.0 to 68.9 years in women [50]. Poland too experienced decline in post-neonatal mortality (available data since the mid-1950s are similar to these of Estonia). We suggest that the underlying reasons for the secular trend in growth in the post-war period in all above-mentioned countries can be ascribed to improved medical conditions during pubertal growth.

Current findings may seem at odds with those of several large-scale longitudinal studies performed in developing countries [10, 51–53], which have usually found that adult size is mainly determined by the growth conditions during the first 3 years of life (but see Ref. 54). We can see at least two mutually non-exclusive explanations for this discrepancy.

First, the nature of chronic environmental stressors [54], including pathogenic infections in northern Europe versus tropics may be qualitatively distinct [55], making comparison of the results of the current study with those collected in low latitudes problematic. For example, high childhood mortality in Africa [56] and poor countries elsewhere [4] is often associated with taller adults, which suggests that mortality selection dominates growth stunting, the opposite of what is found in the rest of the world [56]. Such differences in environmental constraints and selection regimes may well lead to regional differences with respect to sensitivity of growth to external exposures. We suggest that such regional differences might explain why, for instance, secular increase in height for Czech children between 1800 and 2001 could be ascribed to increased growth velocities around the period of pubertal growth spurt [19], contrary to what has been reported for the tropics [57].

Second, the time-window of this study captured a period of particularly abrupt epidemiological transition as demonstrated by the absence of correlation between infant mortality in the birth year and infant mortality at age 11. Such rapid changes in the pathogen load may be uncommon in countries experiencing different stages of epidemiological transition. Indeed, it has been claimed that there is no evidence that the general environment experienced by adolescents in developing countries differs from that of pre-adolescents (Ref. [54], see also Ref. [42]). In situations where infant mortalities in the year of birth and during the pubertal growth spurt correlate strongly and positively, distinguishing the effect of external factors on growth at different ages would be difficult [42]. Furthermore, we are not aware of any other study that had explicitly compared the effects of the growth environment at birth versus at puberty upon adult size and performance.

The limitation of cross-sectional studies of adolescents, including ours, is the inevitable underestimation of the effects of early growth conditions: those suffering most due to harshness of the early environment are under-represented in a final sample due to selective mortality and dropping out from school. The size of this lost segment varies under different ecological/socioeconomic settings [4] and its estimation, wherever possible, would be important. Nevertheless, we doubt that a sizeable proportion of secular increase in growth in developed countries could be ascribed to selective disappearance of small pre-adolescent children. Rather, the historical samples demonstrate increased growth rates during puberty (e.g. Refs. [19, 49]).

Absence of secular increase in leg length is very consistent with the argumentation provided above, assuming that leg and trunk length are affected by exposures at different ages. There is ample evidence for the latter (reviewed by Refs. [22, 23]). For instance, an extensive longitudinal study of the British national birth cohort of 1946 showed that leg length was associated positively with breastfeeding and energy intake at four years while trunk length was associated negatively with serious illness in childhood, but not with dietary data [58].

Our findings contribute to an ongoing debate about whether the secular trend in stature is primarily determined by increases in leg or trunk length (reviewed by Ref. [59]). We suggest that a universal answer for this question probably does not exist. We propose that in situations where the improvement of growth conditions is primarily determined by rapidly increasing efficiency of pathogen control during the pubertal growth period, an increase in trunk length may drive the secular trends in height. Conversely, in situations where the disease environment remains stable throughout the growth period, nutritional and/or other socioeconomic factors may be more important. In our study, both leg and trunk lengths were negatively affected by the number of siblings while parental SEP affected only leg but not trunk length (Figs 1 and 3). As a result, the stature was affected by both sibling number and parental SEP, although notably, both effects were weaker than the secular trend (Fig. 1).

Trade-offs between the number of children in a family and various indices of their performance have been well established in modern low fertility, low mortality populations (reviewed by Refs. [5, 60]). These findings are consistent with both life-history theory and simple models of household resource dilution which posit that, all else being equal, individuals raised in larger families are disadvantaged due to dilution of finite amount of parental investment between siblings. Additionally, increasing number of siblings may impinge on their quality via increased pathogen transmission [42, 43, 61]. In this study, the number of siblings affected all three measures of length (Fig. 1), but none of the other measured variables. Furthermore, the effect of sibling number upon height was rather small as the girls from families with one or two children were only 0.8 cm taller than the girls with three or more siblings (Fig. 3A). A much stronger effect of sibling number on height was found by Lawson and Mace [62] among a large cohort of children born in 1991 and 1992 in Avon, UK. In that study, 10-year-old children in one child families were more than 3 cm taller than children who had three or more siblings. Unfortunately, the Avon data are not directly comparable to these of this study because it is not known to what extent compensatory growth affects the stature of children by adolescence in UK. Nonetheless, one might speculate that in modern welfare societies where the effect of pathogens on growth is curtailed, the effect of resource limitation becomes more prominent.

Parental SEP affected only height and sitting height, the effect being slightly larger than the effect of sibling number (Figs 1 and 3B). Girls whose parents were unskilled manual workers were on average 1.3 cm shorter than those girls whose parents worked in professions requiring tertiary or secondary education. Qualitatively, this finding compares favourably with many previous ones (reviewed by Ref. [63]). However, the magnitude of the effect of SEP on height was much smaller than in most other studies where the maximum difference between strata usually exceeded 3 cm (Table 1 in Ref. [63]). The finding of the current study, that SEP affected leg length but not sitting height suggests that parental assets had stronger effect on early than pubertal growth. Interestingly, the situation was reversed with the impact of urban versus rural origin. Urban girls had longer trunks than rural ones, which suggest that the beneficial effects associated with urbanisation were limited to the period of pubertal growth. Rural girls, on the other hand, were stronger (Fig. 1), which can be most likely explained by imposed manual work load.

### Head size

Presence of a secular trend in cranial volume is particularly interesting because the brain obtains 95% of its final size by the age of six [64]. Nevertheless, an adolescent growth spurt in head size, involving neural tissue, is also documented [65] and the amount of white matter (glia and myelinated axons) in the brain generally increases throughout childhood and adolescence [41, 64]. Increase of cranial volume with age can be also detected in the current dataset (Table 1).

Given that cranial volume, brain size and IQ are intercorrelated [27], our finding that head size covaried with infant mortality in the period of the growth spurt provides novel (though indirect) evidence in favour of the hypothesis that a secular trend in IQ, i.e. the Flynn effect, may be caused by declining rates of infection [14]. At that, we stress the importance of the timing of the impact of disease with respect to pubertal growth: we found no evidence that infant mortality in the birth year correlated with head size, while growth conditions during puberty were important.

In this context, we wish to stress the importance of distinguishing the effects of nutrition from these of disease. Although disease and malnutrition are in mutual feedback during growth, we find the claim that the process of reduction in disease can be subsumed under improvements in nutrition (because the effect of diseases on growth is to reduce the absorption of nutrients) [15] incorrect. For instance, well-nourished children in developing countries frequently suffer from respiratory infections [66] including pneumonia and tuberculosis [67]. In this study, the heads of girls around the age of 17 increased by ∼40 cm^3^ during the 16-year period despite deterioration in the quality of pre- and postnatal nutrition after 1941. Secular trends in head size have been observed in Europe, Asia and among US blacks and whites (reviewed by Ref. [68]). We see no reason why such trends could not be, at least partially, responsible for the secular increase in cognitive abilities.

## CONCLUSIONS AND IMPLICATIONS

All the measured variables have been shown to contribute to current and future performance [3, 29, 57], with potential effects on fitness via both viability [29] and fecundity selection [57], including effects on offspring quality [11] and timing of reproduction [2]. The positive associations between body and lung size [30] and muscular strength [31] versus general health and well-being (reviewed by Refs. [7, 8, 23]) as well as income [10] are also well established. The finding that measured anthropometric traits (with a single exception of leg length) were affected by growth conditions during puberty but not by conditions at birth has implications for a number of fields:

1. Epidemiology: Large-scale epidemiological studies (e.g. Refs. [10, 39, 40]) have so far concentrated on effects of foetal and post-neonatal growth condition upon adult size, health and human capital. We suggest that paying equal attention to growth conditions at puberty would reveal new and interesting patterns about how resource limitation and the disease environment affect adult outcomes.
2. Paediatrics: Recognizing that the period of the pubertal growth spurt is particularly sensitive to the disease environment should aid to focus increased attention of child health care programmes to that particular period (see also Ref. [41]).
3. Cognitive science: In search of exposures affecting brain development, moving the focus from the foetal and postnatal period to the period of puberty, and distinguishing the effect of disease from that of nutrition may offer congruent explanations for secular trends in cognitive abilities. Such an approach may be also useful for explaining global variation in cognitive abilities.
4. Economic history: Anthropometric measures are increasingly used in historical studies to understand the impact of environmental factors on the living conditions of individuals and populations. We propose that shifting the time frame from conditions during birth to conditions during puberty (and comparing the conditions in these periods) would increase the explanatory power of such studies.
5. Human life-history theory and evolutionary medicine: Recognizing that the effect of the disease environment encountered during puberty can affect adult phenotype more strongly than resource limitation via sibling competition or parental assets helps to understand the relative importance of different selective forces in formation of life-history trade-offs in different ecological settings. For instance, it would be interesting to test whether the effect of the disease environment on adult phenotype and performance is equally strong in the periods preceding and following major medical innovations occurring in the middle of the 20th century. Comparative anthropometric studies in areas with different disease environments and resource availability [23] too, would benefit from distinguishing the effects of disease from those of resource limitation at different ages.

## SUPPLEMENTARY DATA

Supplementary data is available at *EMPH* online.

Supplementary Data

Supplementary Data
