# Supplementary material for: Why did children grow so well at hard times? The ultimate importance of pathogen control during puberty
Source: Evol Med Public Health. 2015 Jul 20;2015(1):167–78. doi: 10.1093/emph/eov017 (PMC4530472; doi:10.1093/emph/eov017)
Supplement: Supplementary Data [file supp_2015_1_167__index.html]

Why did children grow so well at hard times? The ultimate importance of pathogen control during puberty — Supplementary Data 

# Why did children grow so well at hard times? The ultimate importance of pathogen control during puberty

## Supplementary Data

files

- Supplementary Data - docx file
