# Supplementary material for: Why did children grow so well at hard times? The ultimate importance of pathogen control during puberty
Source: Evol Med Public Health. 2015 Jul 20;2015(1):167–78. doi: 10.1093/emph/eov017 (PMC4530472; doi:10.1093/emph/eov017)
Supplement: Supplementary Data [file supp_eov017_SI.docx]

Supplementary information. Comparison of the effects of birth date and disease burden at birth and at age 11 (assessed on the basis of yearly infant mortality) on anthropometric parameters of adolescent girls in multiple regressions. N = 1475 for all variables. F- and p-values are given for all tested variables, standardized regression coefficients (β) are given for significant predictors only. Empty cells indicate variables not tested in a particular model. SEP: parental socioeconomic position, urban denotes urban vs rural origin. mort0: infant mortality at year of birth, mort11: infant mortality in a year when the girls were 11 years old.

| height | model with MORT0 | | | model with MORT11 | | | model without birth date | | |
| --- | --- | --- | --- | --- | --- | --- | --- | --- | --- |
| effect | F | p | β (SE) | F | p | β (SE) | F | p | β (SE) |
| siblings | 7.3 | 0.007 | -0.07 (0.03) | 7.3 | 0.007 | -0.07 (0.03) | 7.1 | 0.007 | -0.07 (0.03) |
| SEP | 12.0 | 0.0006 | 0.10 (0.03) | 7.3 | 0.007 | 0.10 (0.03) | 12.4 | 0.0004 | 0.10 (0.03) |
| age | 17.8 | 0.00003 | 0.11 (0.03) | 14.2 | 0.0002 | 0.10 (0.03) | 16.2 | 0.00006 | 0.11 (0.03) |
| birth date | 23.9 | <0.00001 | 0.13 (0.03) | 0.4 | 0.543 |  |  |  |  |
| mort0 | 1.3 | 0.256 |  |  |  |  | 1.3 | 0.830 |  |
| mort11 |  |  |  | 4.4 | 0.002 | -0.19 (0.09) | 26.7 | <0.00001 | -0.13 (0.03) |

| sitting height | model with MORT0 | | | model with MORT11 | | | model without birth date | | |
| --- | --- | --- | --- | --- | --- | --- | --- | --- | --- |
| effect | F | p | β (SE) | F | p | β (SE) | F | p | β (SE) |
| siblings | 4.5 | 0.035 | -0.05 (0.03) | 4.2 | 0.041 | -0.05 (0.03) | 4.2 | 0.040 | -0.05 (0.03) |
| urban | 11.3 | 0.0007 | 0.10 (0.03) | 12.8 | 0.0004 | 0.10 (0.03) | 13.6 | 0.0002 | 0.10 (0.03) |
| age | 40.3 | <0.00001 | 0.17 (0.03) | 34.4 | <0.00001 | 0.17 (0.03) | 36.3 | <0.00001 | 0.16 (0.03) |
| birth date | 56.0 | <0.00001 | 0.20 (0.03) | 0.05 | 0.820 |  |  |  |  |
| mort0 | 1.8 | 0.075 |  |  |  |  | 0.2 | 0.678 |  |
| mort11 |  |  |  | 6.1 | 0.013 | -0.23 (0.09) | 59.3 | <0.00001 | -0.20 (0.03) |

| leg length | model with MORT0 | | | model with MORT11 | | | model without birth date | | |
| --- | --- | --- | --- | --- | --- | --- | --- | --- | --- |
| effect | F | p | β (SE) | F | p | β (SE) | F | p | β (SE) |
| siblings | 4.2 | 0.040 | -0.05 (0.03) | 4.2 | 0.039 | -0.06 (0.03) | 4.1 | 0.044 | -0.06 (0.03) |
| SEP | 7.9 | 0.005 | 0.08 (0.03) | 9.1 | 0.003 | 0.08 (0.03) | 7.6 | 0.006 | 0.08 (0.03) |
| birth date | 0.4 | 0.521 |  | 2.0 | 0.148 |  |  |  |  |
| mort0 | 0.4 | 0.520 |  |  |  |  | 0.2 | 0.640 |  |
| mort11 |  |  |  | 2.9 | 0.090 |  | 1.0 | 0.311 |  |

| cranial volume | model with MORT0 | | | model with MORT11 | | | model without birth date | | |
| --- | --- | --- | --- | --- | --- | --- | --- | --- | --- |
| effect | F | p | β (SE) | F | p | β (SE) | F | p | β (SE) |
| age | 12.2 | 0.0005 | 0.09 (0.03) | 8.4 | 0.004 | 0.09 (0.03) | 10.1 | 0.001 | 0.08 (0.03) |
| birth date | 52.4 | <0.00001 | 0.19 (0.03) | 0.0 | 0.864 |  |  |  |  |
| mort0 | 0.1 | 0.791 |  |  |  |  | 1.2 | 0.280 |  |
| mort11 |  |  |  | 3.8 | 0.049 | -0.18 (0.09) | 57.6 | <0.00001 | -0.20 (0.03) |

| Lung capacity | model with MORT0 | | | model with MORT11 | | | model without birth date | | |
| --- | --- | --- | --- | --- | --- | --- | --- | --- | --- |
| effect | F | p | β (SE) | F | p | β (SE) | F | p | β (SE) |
| mass | 240.0 | <0.00001 | 0.37 (0.02) | 234.6 | <0.00001 | 0.38 (0.02) | 233.4 | <0.00001 | 0.37 (0.02) |
| birth date | 54.3 | <0.00001 | 0.18 (0.02) | 7.3 | 0.007 | 0.22 (0.08) |  |  |  |
| mort0 | 1.5 | 0.224 |  |  |  |  | 6.9 | 0.009 | -0.06 (0.02) |
| mort11 |  |  |  | 0.3 | 0.610 | - | 52.6 | <0.00001 | -0.17 (0.02) |

| handgrip | model with MORT0 | | | model with MORT11 | | | model without birth date | | |
| --- | --- | --- | --- | --- | --- | --- | --- | --- | --- |
| effect | F | p | β (SE) | F | p | β (SE) | F | p | β (SE) |
| urban | 10.5 | 0.001 | -0.08 (0.03) | 9.1 | 0.003 | -0.08 (0.03) | 9.0 | 0.003 | -0.08 (0.03) |
| age | 28.0 | <0.00001 | 0.13 (0.03) | 24.4 | <0.00001 | 0.13 (0.03) | 24.9 | <0.00001 | 0.12 (0.03) |
| mass | 221.7 | <0.00001 | 0.36 (0.02) | 218.9 | <0.00001 | 0.36 (0.02) | 218.8 | <0.00001 | 0.36 (0.02) |
| birth date | 55.4 | <0.00001 | 0.19 (0.03) | 0.2 | 0.653 |  |  |  |  |
| mort0 | 1.1 | 0.303 |  |  |  |  | 0.1 | 0.764 |  |
| mort11 |  |  |  | 3.1 | 0.079 |  | 57.4 | <0.00001 | -0.19 (0.03) |
